# Crystal structure of the flexible tandem repeat domain of bacterial cellulose synthesis subunit C

**DOI:** 10.1038/s41598-017-12530-0

**Published:** 2017-10-12

**Authors:** Shingo Nojima, Ayumi Fujishima, Koji Kato, Kayoko Ohuchi, Nobutaka Shimizu, Kento Yonezawa, Kenji Tajima, Min Yao

**Affiliations:** 1 Graduate school of Life Science, Hokkaido University, Sapporo, Hokkaido, 060-0810 Japan; 2 Faculty of Advanced Life Science, Hokkaido University, Sapporo, Hokkaido, 060-0810 Japan; 3 Photon Factory, Institute of Materials Structure Science, High Energy Accelerator Research Organization (KEK), Tsukuba, Ibaraki, 305-0801 Japan; 4 Faculty of Engineering, Hokkaido University, Sapporo, Hokkaido, 060-8628 Japan

## Abstract

Bacterial cellulose (BC) is synthesized and exported through the cell membrane via a large protein complex (terminal complex) that consists of three or four subunits. BcsC is a little-studied subunit considered to export BC to the extracellular matrix. It is predicted to have two domains: a tetratrico peptide repeat (TPR) domain and a β-barrelled outer membrane domain. Here we report the crystal structure of the N-terminal part of BcsC-TPR domain (Asp24–Arg272) derived from *Enterobacter* CJF-002. Unlike most TPR-containing proteins which have continuous TPR motifs, this structure has an extra α-helix between two clusters of TPR motifs. Five independent molecules in the crystal had three different conformations that varied at the hinge of the inserted α-helix. Such structural feature indicates that the inserted α-helix confers flexibility to the chain and changes the direction of the TPR super-helix, which was also suggested by structural analysis of BcsC-TPR (Asp24–Leu664) in solution by size exclusion chromatography-small-angle X-ray scattering. The flexibility at the α-helical hinge may play important role for exporting glucan chains.

## Introduction

Cellulose is the most abundant biopolymer on Earth and is widely used, for example, in paper, T-shirts, and wooden furniture^1^. Cellulose is synthesized by plants, algae, some kinds of bacteria, and even some animals^2–6^. Cellulose produced by some Gram-negative bacteria is called bacterial cellulose (BC) and has different properties to other cellulose in several respects. First, it usually exists highly pure state compared with plant cellulose which is associated with lignin and hemicellulose in the plant cell wall^7^. Second, it has a Young’s modulus (>15 GPa) more than three times higher than that of plant cellulose^8^. Third, it can hold water and form a gel^7^. Because of these properties, BC is used in diaphragms, tubings that may function as blood vessels, and burn wound dressings^9–13^.

BC-producing bacteria have a cellulose synthase operon in their genome. In *Gluconacetobacter xylinum*, the cellulose synthase operon codes three or four proteins: BcsA, BcsB (or BcsAB), BcsC, and BcsD^14–17^. These proteins form a large BC synthesis complex, termed the terminal complex. BcsA and BcsB are located on the inner membrane. BcsA is required to catalyze synthesis of the glucan chain and BcsB is essential for BcsA activation and may guide the glucan chain toward outer membrane by its periplasmic domain^15,18,19^. The crystal structure of the catalytically inactive BcsA-B complex derived from *Rhodobacter sphaeroides* was reported^19^. BcsD is considered as a periplasmic protein. BcsD-deleted *G. xylinum* can produce only 9.6% of the amount of cellulose of the wild type, and the morphology of BC changes, showing that BcsD functions to crystallize glucan chains^15,20^. The crystal structure of BcsD revealed that its octamer forms a cylindrical shape and four glucan chains can pass through the inside of the cylinder^20^.

BcsC is thought to function as the exporting pore of BC, as one study showed that BcsC-deleted *G. xylinum* could not produce BC^14^. BcsC consists of about 20–30 residues of signal peptides, followed by about 750–900 residues of periplasmic domain of tetratrico peptide repeat (BcsC-TPR), and about 380 residues of the outer membrane domain of the β-barrel (BcsC-C)^15,21^. The TPR domain consists of tandem repeats of 34 amino acids. Its degenerate consensus sequence was defined by a pattern of small and large hydrophobic amino acids: highly conserved residues at positions 8 (Ala or Gly), 20 (Ala), and 27 (Ala), and large hydrophobic amino acids at positions 4, 7, 11, and 24. Moreover, position 32, located in the turn between two TPR motifs, usually contains helix-breaking residues like proline^22^ (Fig. S1). The unit motif of TPR is composed of two anti-paralleled α-helices connected by a turn. The multiple TPR motifs assemble into a super-helix that has a convex and a concave face. Most TPR domains have their interaction site on the concave face, but some TPR domains can bind other proteins on their convex face.

BcsC-TPRs are considerably conserved at the consensus residues of the TPR motif, but chain lengths vary among Gram-negative bacteria (Fig. S2). Moreover, AlgK and PgaA, which are located at the outer membrane and are essential proteins for exporting polysaccharides, alginate, and poly-β-1,6-GlcNAc, respectively, also contain a TPR domain^23–25^ and are classified as the same family as BcsC. Thus, the TPR domain is believed to be important for exporting polysaccharides. However, the detailed structure and/or function of the BcsC-TPR domain have not yet been elucidated. In this report, we determined the crystal structure of the N-terminal six TPR motifs in BcsC (BcsC-TPR(N6), Asp24–Arg272) from *Enterobacter* CJF-002. We found a non-TPR α-helix was inserted in the middle of TPR motifs in this structure. Five BcsC-TPR(N6) molecules in an asymmetric unit showed three conformations. A comparison of these three conformations indicated that the interrupted α-helix was found to induce a conformational change in BcsC-TPR(N6). Furthermore, we analysed the structure of BcsC-TPR (Asp24–Leu664) in solution by size exclusion chromatography-small-angle X-ray scattering (SEC-SAXS). An envelope model calculation showed a helical elongated extension of the structure of BcsC-TPR(N6), suggesting that the inserted α-helix confers flexibility to changes in the direction of the TPR super-helix. Such flexibility is possibly important for its cellulose exporting function.

## Results

### Stable region of BcsC-TPR domain

First, full length of BcsC-TPR domain (Asp24–Arg784) of *Enterobacter* CJF-002 were expressed by *E. coli* BL21 (DE3). BcsC-TPR domain was well purified by nickel and size exclusion chromatography. However, purified sample was sequentially degraded during purification and crystallization. Therefore, we tried to find the stable parts of BcsC-TPR domain using the limited proteolysis analysis. Two main fragments were obtained by limited trypsinolysis (Fig. S3) and the molecular weights of these fragments were determined to be 27,430 Da and 43,803 Da by MALDI-TOF-MS. N-terminal amino acid sequence analysis of these fragments indicated that these fragments started from sequences of SHMDEPTA and QAVNAXQQ, respectively. The N-terminal sequence and the molecular weight suggested that these two fragments were residues Asp24–Arg272 and Glu295–Arg695 of BcsC-TPR domain. Based on these results, we constructed several fragments of BcsC-TPR domain, and successfully purified BcsC-TPR (Asp24–Arg272) and BcsC-TPR (Asp24–Leu664).

### Overall structure of BcsC-TPR(N6)

**Figure 1:**
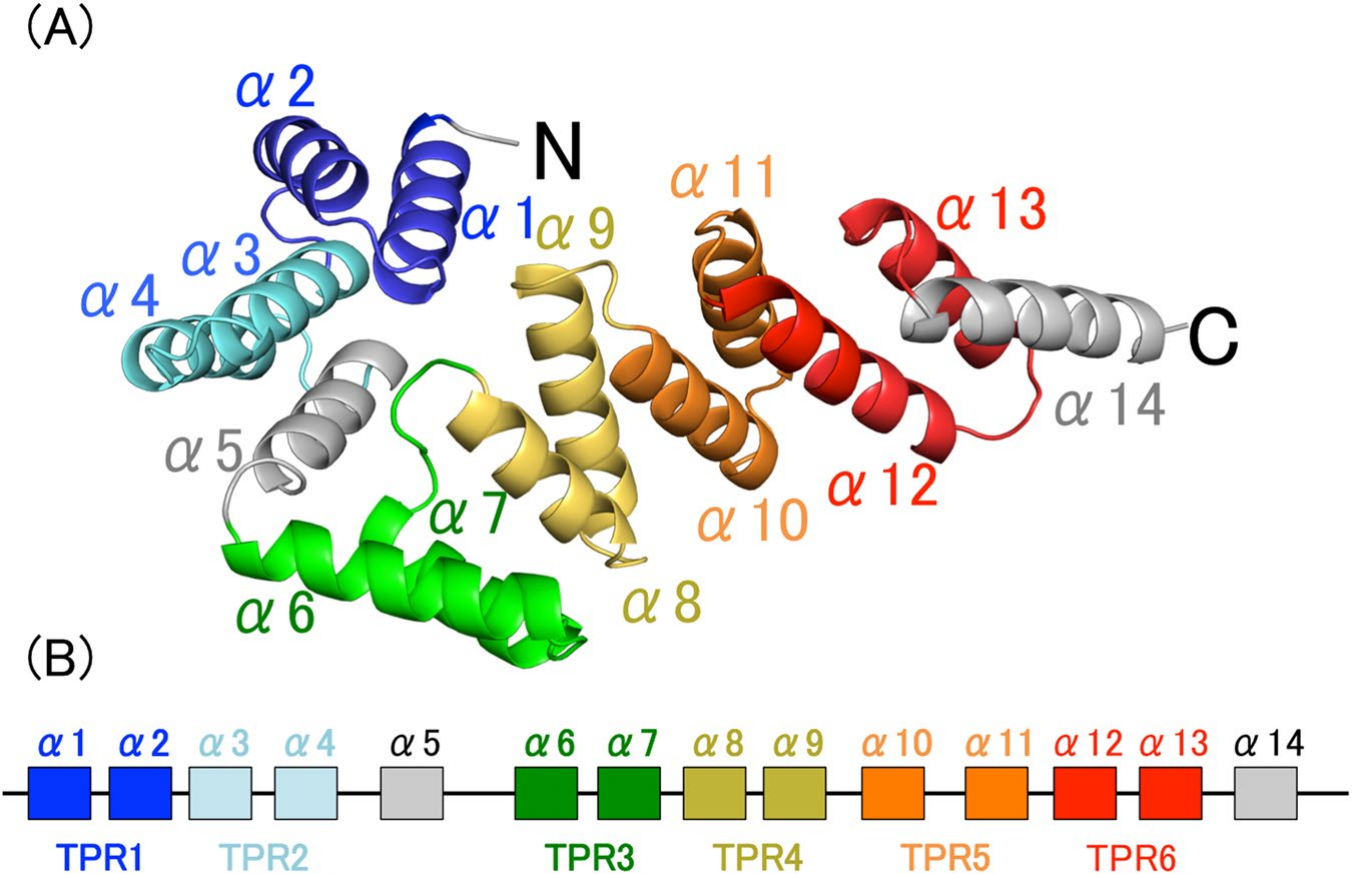
The structure of BcsC-TPR(N6). (**A**) BcsC-TPR(N6) is composed of six TPR motifs (colored blue, light blue, green, yellow, orange, and red) and two unpaired α-helices (gray). (**B**) Schematic diagram of the secondary structure of BcsC-TPR(N6). The boxes indicate α-helices and the lines indicate turn parts. The color scheme is the same as (**A**).

**Figure 2:**
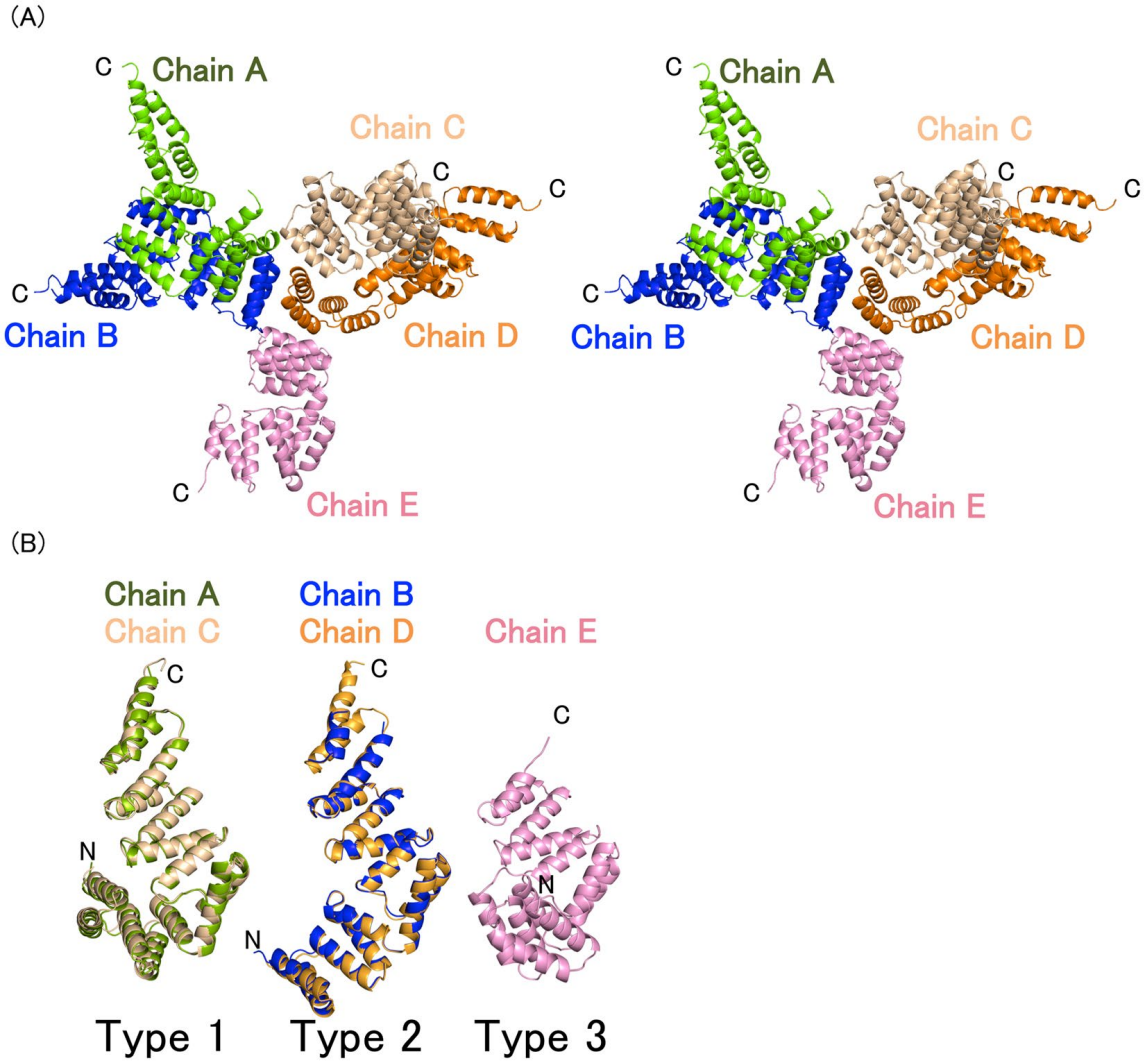
The conformations of BcsC-TPR(N6). (**A**) Stereo view of five structures of BcsC-TPR(N6) in an asymmetric unit. (**B**) Five structures of BcsC-TPR(N6) formed three conformations (type 1, 2, and 3). BcsC-TPR(N6)s are shown in different colors (chain A: Green, chain B: Blue, chain C: wheat, chain D: orange, chain E: pink).

Overexpressed BcsC-TPR (Asp24–Arg272) was purified and crystallized. The crystal structure of BcsC-TPR (Asp24–Arg272) was determined at a resolution of 3.27 Å (Figs 1A and S4). There were five molecules in the asymmetric unit (Fig. 2A) and the Matthew’s coefficient was calculated to be 3.9 Å^3^ Da^−1^ with a solvent content of 68.5%^26^. The five molecules (chain A–E) in the asymmetric unit were not fully built because of the poor electron density in the N- or C-terminal region. Constructed regions were residues Pro26–Asp268 for chain A, residues Asp24–Pro230 and the main chain of residues Ser248–Gln258 for chain B, residues Pro26–Ala270 for chain C, residues Thr27–Ala267 for chain D, and residues Thr27–Gln225 for chain E.

BcsC-TPR(Asp24–Arg272) is a helical structure formed by 14 α-helices (α1–α14) (Fig. 1). Among them, six pairs of helices, α1–α2 (Ala28–Thr42 and Glu45–Ile58), α3–α4 (Pro63–Gln76 and Asn79–Leu92), α6–α7 (Ala111–Thr126 and Val129–Phe140), α8–α9 (Asp148–Lys159 and Arg163–Arg177), α10–α11 (Ala182–Gly194 and Asp198–Lys210) and α12–α13 (Arg215–Arg228 and Asp233–Val245) form six TPR motifs (TPR1: Ala28–Asn62, TPR2: Pro63–Ser96, TPR3: Ala111–Gly147, TPR4: Asp148–Asn181, TPR5: Ala182–Ala214, TPR6: Arg215–Gly249), while α5 (Ala97–Leu108) and α14 (Thr251–Gln265) do not belong to TPR motifs (Fig. 1B). We, therefore, referred BcsC-TPR (Asp24–Arg272) as BcsC-TPR(N6).

### BcsC-TPR(N6) consists of two TPR domains connected by a hinge

**Figure 3:**
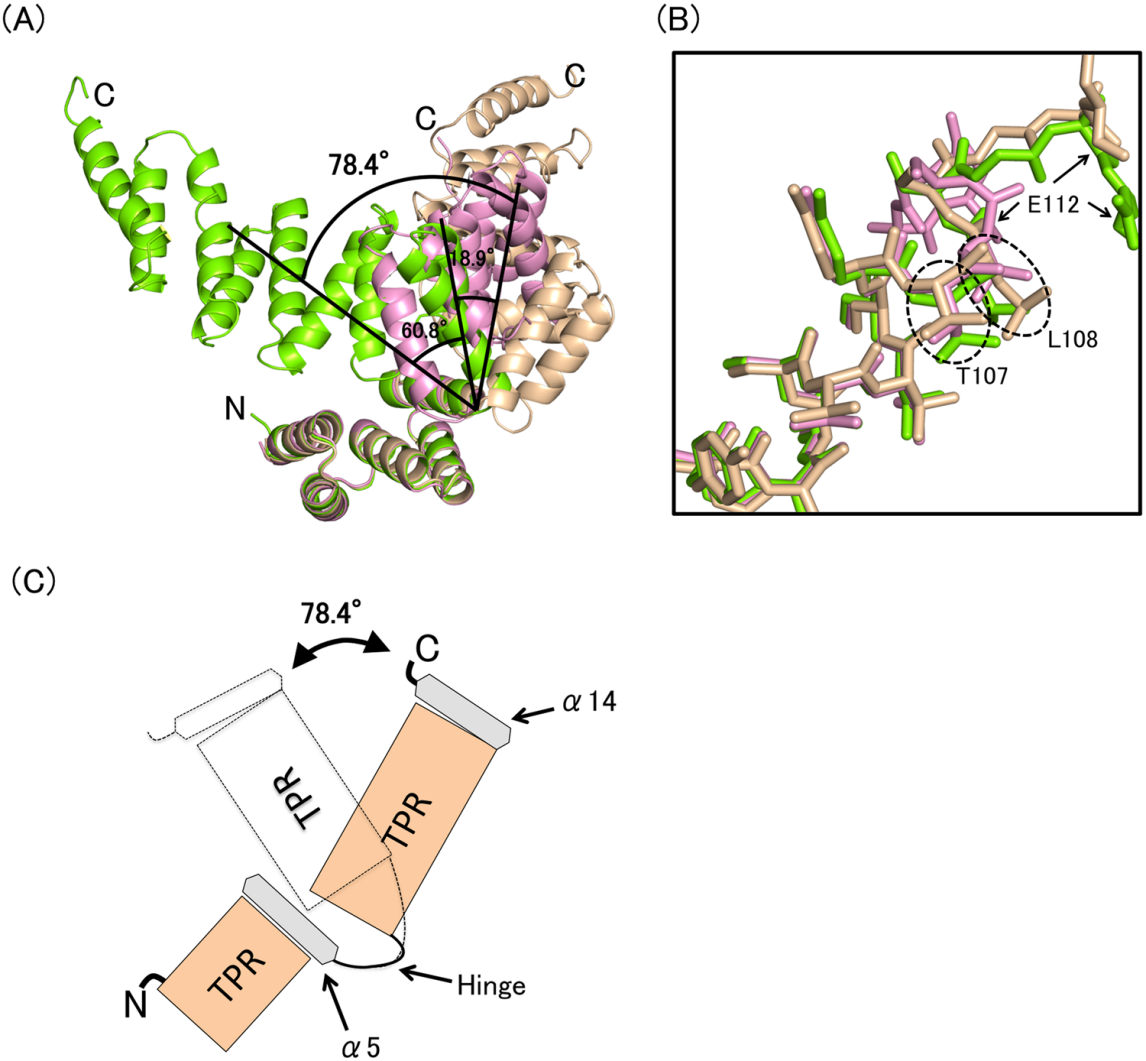
Structural comparison of BcsC-TPR(N6)s. (**A**) Superposition of three type chains on α1–α5. Black angles indicate the angle of the Cα of Ala219 (Chain A) and Leu108 (Chain A), Leu108 (Chain A) and Ala219 (Chain D). (**B**) Zoomed in and shown in stick format around the turn of inserted region. (**C**) Schematic image of the flexible region.

A comparison of five monomers in the asymmetric unit showed that they can be classified into three different conformations: chains A and C were type 1 (root mean square deviation (r.m.s.d.) was 0.74 Å for 243 Cα atoms), chains B and D were type 2 (r.m.s.d. was 0.38 Å for 204 Cα atoms), and chain E was type 3 (Fig. 2B). The conformations of these three types were different at the turn region between α5 and α6. When N-terminal helices (α1–α5) of five molecules were superposed (r.m.s.d. = 0.38–0.50 Å over 80 residues), the C-terminal half (α6–α11) of each chain extended in a different direction with an angular difference of 18.9°–78.4° (Fig. 3A). Similarly, each N-terminal half (α1–α5) showed directional difference when the C-terminal half (α6–α11) was superposed (r.m.s.d. = 0.71–1.37 Å over 99 residues) (Fig. S5). The C-terminal two helices (α12–α13) were not included for this calculation since this region was not built for chain E. These results indicate that BcsC-TPR(N6) consists of two structural domains, an N-terminal TPR domain (α1–α5), and a C-terminal TPR domain (α6–α11), connected by a hinge (Fig. 3B and C) at Thr107–Ala111. The residues of Ala97–Gly110 which are composed of α5 (Ala97–Leu108) and the loop (Ser109–Gly110) do not belong to the TPR motif. The α5 interacted with TPR2 and formed a similar conformation among type 1–3. Thus, α5 seems to interfere with the formation of the super-helix between TPR2 and TPR3 through the interaction with TPR2. Based on the superposition of types 1 and 3, the C-terminal super-helix changes its screw axis by a conformational change at the loop of Ser109–Gly110. Collectively, the inserted non-TPR region can be considered to play two important roles: covering the interaction region of the upstream TPR motif to separate from the downstream TPR motif, and changing the direction of the downstream super-helix at the loop.

### SAXS data reveal the architecture of the BcsC-TRP

**Figure 4:**
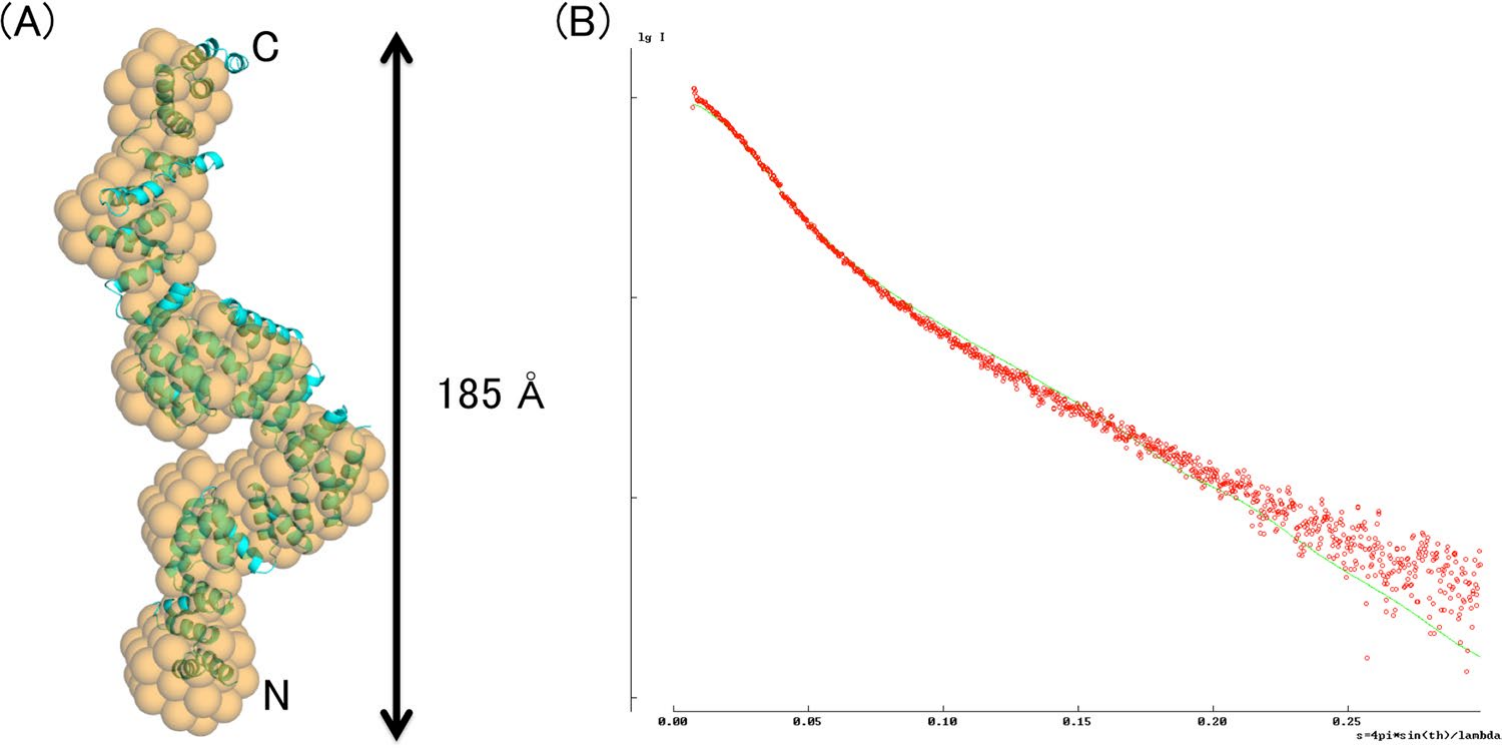
The structural analysis of BcsC-TPR in solution by SEC-SAXS. (**A**) The SAXS envelope (orange) and fitted structure of BcsC-TPR (cyan). Orange spheres indicate the SAXS envelope calculated from random dummy atoms. Cyan cartoon shows BcsC-TPR super-helices modeled by fitting a poly-Ala structure of BcsC-TPR(N6) as the TPR unit on the SAXS envelope. (**B**) Comparison between the calculated one-dimensional scattering intensity data (green line) of BcsC-TPR model and the measured one-dimensional scattering intensity data of SEC-SAXS (red dots).

Based on the result of the limited proteolysis analysis, we obtained a stable sample of BcsC-TPR (containing Asp24–Leu664 residues), and tried to determine the crystal structure. However, we only obtained crystals that diffracted to 7–8 Å resolution and could not determine the structure by molecular replacement, using the structures of BcsC-TPR(N6) or other known TPR domains as search models. In order to investigate the structural information of the BcsC-TPR in solution, we performed a SEC-SAXS experiment. R_g_ values were 51.2 (+/−0.8) Å for Guinier analysis and 51.3 Å for P(r), respectively. This value is approximately twofold larger than the calculated envelope R_g_ value (25.3 Å) of the BcsC-TPR(N6) structure. The molecular weight from SEC-MALS (multi-angle static light scattering) was 71.4 kDa, almost identical to that of the Porod volume of 72.1 kDa. A plot of the P(r) function showed an elongated shape with a *D*_max_ = 185 Å which is also more twice that of the BcsC-TPR(N6) structure (Fig. 4A). Finally, we calculated an envelope model with a matched parameter of χ^2^ volume of 1.906 using one-dimensional scattering intensity data of SEC-SAXS. As shown in Fig. 4, the model shows a helical elongated extension of BcsC-TPR(N6) structure.

## Discussion

In this study, we determined the structure of BcsC-TPR(N6), which has six of nineteen TPR motifs in BcsC from *Enterobacter sp*. CJF-002. We compared BcsC-TPR(N6) with other proteins containing a TPR domain by Dali server^27^. Almost all the hit proteins with a high z-score only matched with α1–α5 of BcsC-TPR(N6). To focus on the characteristics of BcsC containing long TPR domains, we picked up two proteins that had a distinctively large number of TPR motifs (more than eight) from the results of Dali server: *O*-GlcNAc transferase, OGT^28,29^ (containing 13 TPR motifs, Z-score = 12.3) and an interferon-induced protein with tetratricopeptide repeats, IFIT5^30^ (containing nine TPR motifs, Z-score = 10.8).

**Figure 5:**
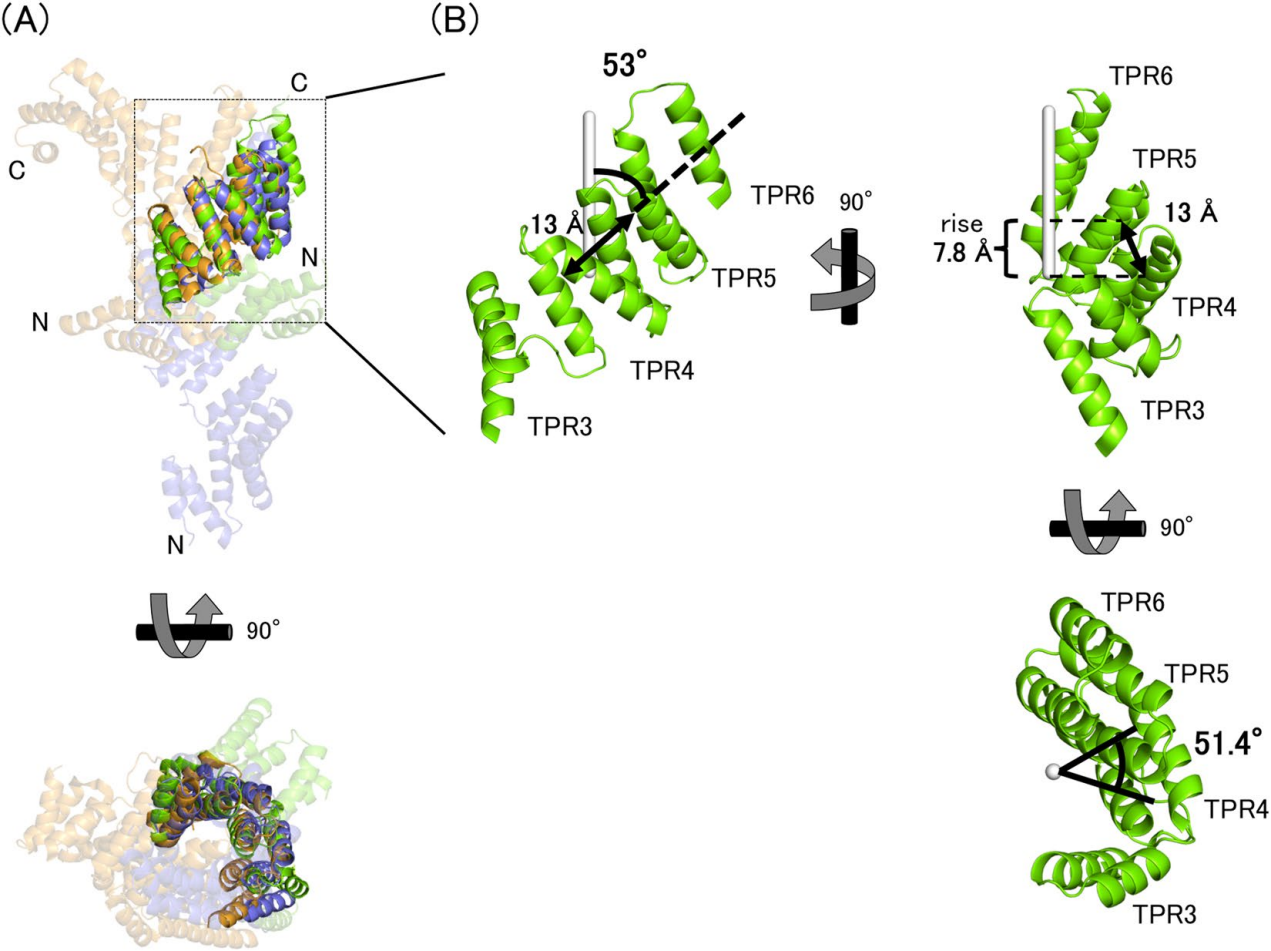
Structural comparison of BcsC-TPR(N6)s. (**A**) Comparing TPR super-helices of different proteins. Green, BcsC-TPR(N6); blue, OGT; orange, IFIT5. Opaque cartoons indicate the TPR super-helices that are superposed. TPR super-helices are almost same structure. (**B**) The typical parameters of TPR super-helix. Green, SH2 of BcsC-TPR(N6); white stick, axis of super-helix.

BcsC-TPR(N6) is composed of two TPR super-helices (super-helix 1, SH1: TPR1–TPR2 and superhelix2, SH2: TPR3–6). A comparison of the TPR super-helices of OGT, IFIT5, and BcsC-TPR(N6) showed they had a similar shape. The number of TPR motifs per super-helical turn was about seven with a pitch of 55 Å^28^. One TPR consisted of two α-helices, and the average shift length between neighbour TPR motifs was 13 Å. This shift direction was inclined to the axis of super-helix by about 53°, such that the rise of the super-helix per a TPR motif was 7.8 Å (=13 × cos53°) and the length of a pitch was 55 Å (=7.8 × 7) (Fig. 5).

**Figure 6:**
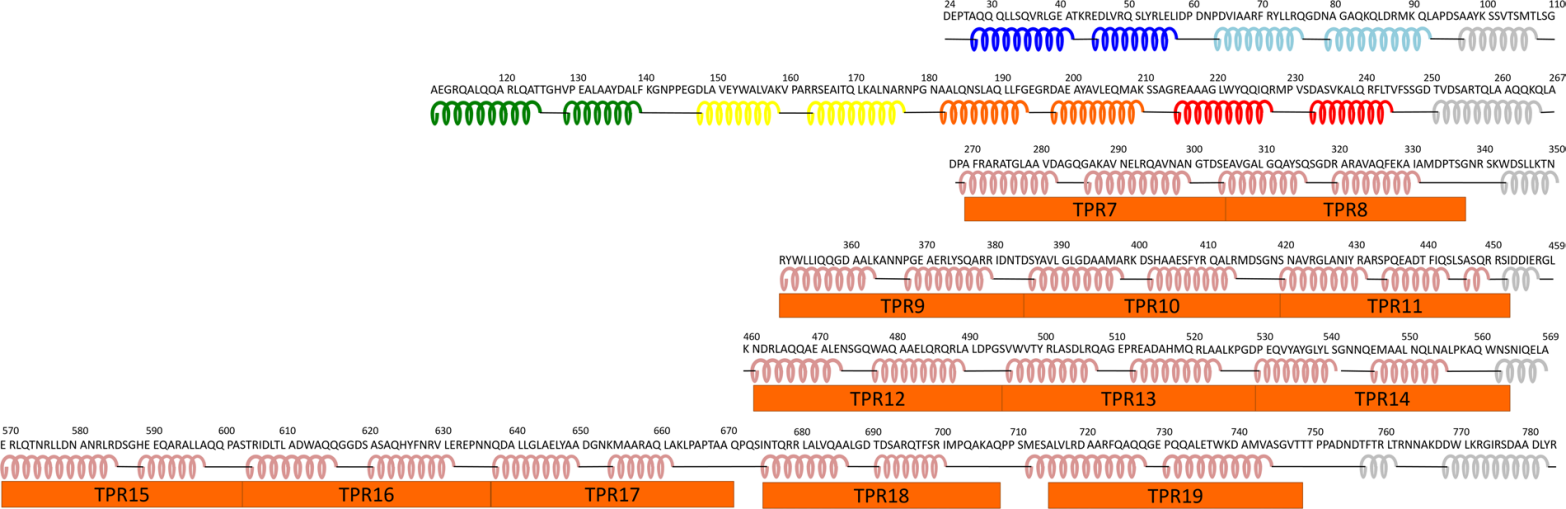
Secondary structure and TPR motifs of BcsC-TPR domain (Asp24–Tyr783). The secondary structure elements are represented by coil (α-helix) and bar (loop), while the TPR motif predicted by TPRpred is represented by orange boxes. The secondary structure of the first two rows (BcsC-TPR(N6): Asp24–Ala267) are based on the crystal structure (colors are same as Fig. 1), and the other regions are based on the secondary structure prediction program (the PSIPRED server).

The secondary structure prediction (PSIPRED server) and TPR prediction (TPRpred server) programs^31–33^ indicated that the whole BcsC-TPR had nineteen TPR motifs including six N-terminal TPR motifs observed in the present analysis. Downstream of every two or three consecutive TPR motifs in BcsC-TPR, there are non-TPR regions of 6–16 amino acid residues (Fig. 6). These non-TPR regions possibly interfere with the interaction of consecutive TPR motifs and change the directions of TPR super-helices. The non-TPR region may also possibly form flexible hinges, as shown in the present analysis. Such flexibility of the TPR domain via breaks was further confirmed by SEC-SAXS analyses. We obtained the SEC-SAXS result of BcsC-TPR (Asp24–Leu664, containing N-terminal 17 TPR motifs) with long sharp, and matched the SAXS envelope model well with a poly-Ala model that was built using the poly-Ala structure of BcsC-TPR(N6) as the TPR unit of TPR super-helices. The χ^2^ volume between the calculated SAXS data of atomic poly-Ala model and the measured SEC-SAXS data of BcsC-TPR was 7.123 (Fig. 4B). Surprisingly, the putative hinge regions (Fig. 6) of the fitted structure were located at the necks of the SAXS envelope, supporting our prediction of the structure of BcsC-TPR. Therefore, we proposed that BcsC-TPR forms six super-helices that have consistent structures connected by five non-TPR regions. These non-TPR regions change the direction of the super-helices and may enable BcsC-TPR to create a structure for transporting nascent cellulose chains.

Next, we focused on homologs of BcsC-TPR and the length of TPR domain, binding partners, and flexibility. OGT has a series of 13 TPR motifs without inserted residues and this TPR domain can bind unfolded protein to *O*-GlcNAcylate at serine/threonine^28,29^.

IFIT5 can bind viral ssRNA to prevent its transcription. Nine TPR motifs of this protein are also separated into two, four, and three TPR motifs by a long linker loop and two α-helices, respectively. These inserted regions break the hyper helix formed by the series of TPR motifs. A unique feature of IFIT5 is that three domains of IFIT5 form the binding pocket of viral ssRNA cooperatively^30^.

In another instance of a long TPR domain, Keiski *et al*. revealed the structure of a TPR-containing protein, AlgK, which belongs to the same family as BcsC. AlgK is a subunit of the alginate synthase complex and plays a role in the assembly of the alginate synthetic complex^34^. AlgK protrudes from the outer membrane to the periplasm, and is composed of 9.5 TPR-like motifs without an inserted α-helix^23^. Compared with AlgK, BcsC-TPR has inserted α-helices and five putative flexible points, and contains twice as much TPR motifs as AlgK.

From these TPR-containing proteins, long TPR domains without any inserted region interact with unfolded proteins like OGT. Long TPR domains with inserted regions form a binding pocket of molecules other than unfolded proteins using flexible hinges like IFIT5. In other words, TPR domains change these lengths and are flexible to bind to specific ligands. Current studies imply that flexibility derived from hinges is a useful property to form interactions, and this should be confirmed by structural analysis of BcsC with glucan chain in the future. The detail function of BcsC has been unclear since BcsC was characterized as a component of TC. We believe our findings are the first breakthrough of the elucidation of exporting mechanism of BcsC, and are the key to clarify the synthetic mechanism of BC which is of great biological and economical importance.

## Methods

### Construction of BcsC-TPR vector and expression

The BcsC-TPR domain (Asp24–Arg784), the stable parts of BcsC-TPR (Asp24–Leu664) and BcsC-TPR(N6) (Asp24–Arg272) were cloned into the NdeI-XhoI site of a modified pET-28a vector (Novagen), in which a *Tobacco etch virus* (TEV) protease recognition site was introduced between the His_6_ tag and the N-terminus of the target protein instead for the thrombin site^35^. Another His_6_ tag was also introduced at the C-terminus of the target protein (N-, C-His tagged BcsC-TPR domain; N-, C-His tagged BcsC-TPR; and N-, C-His tagged BcsC-TPR(N6)). His tags were conjugated at both sides of the target proteins because they were easily fragmented during overexpression and purification, and a second nickel affinity chromatography was needed after the first nickel affinity chromatography to obtain a pure target protein. The primer information is summarized in Table S1.

This plasmid was introduced into *Escherichia coli* BL21 (DE3). The cells were cultivated in 1 L Luria broth medium supplemented with 25 µg mg^−1^ kanamycin at 309 K. When the optical density (OD) at 600 nm of the broth reached 0.5, 100 µM isopropyl β-D-1-thiogalactopyranoside (IPTG) was added then cultivated overnight at 298 K to induce expression. The cells were harvested by centrifugation at 4500 × g for 15 min at 277 K and were resuspended in lysis buffer (50 mM HEPES, pH 8.0; 500 mM KCl; 10% glycerol; 2.5 μg mL^−1^ DNase I; 1 mg mL^−1^ lysozyme).

To obtain selenomethionine-substituted BcsC-TPR(N6) (Se-BcsC-TPR(N6)), the expression host was cultivated in 1× MS medium (100 mL of 10× M9 salt solution (337 mM disodium phosphate 12-hydrate, 220 mM potassium dihydrogen phosphate, 85.5 mM NaCl, and 93.5 mM ammonium chloride in 1 L water, pH 7.2), 0.4 % glucose, 1 mM magnesium sulfate, 0.3 mM calcium chloride, 1 μg biotin, 1 μg thiamin, 1× trace element solution) containing 25 µg mg^−1^ kanamycin at 309 K. When the OD at 600 nm of the medium reached 0.6, 100 mg L^−1^ of L-Lys, L-Thr, L-Ile, L-Leu, L-Val, L-Phe, and 25 mg L^−1^ selenomethionine was added and cultivated at 309 K for 30 min. Subsequently, 100 µM IPTG was added and cultivated overnight at 298 K to induce expression. Cells were harvested by centrifugation at 4500 × g for 15 min at 277 K and resuspended in lysis buffer.

### Purification of native and selenomethionine-substituted BcsC-TPR

Native BcsC-TPR domain, BcsC-TPR and BcsC-TPR(N6) (Asp24–Arg272) were purified using the same protocol. The cells were lysed twice using a French press at 6000 psi and separated into supernatant and precipitate by centrifugation at 40,000 × g for 15 min at 283 K. The supernatant was filtrated through 0.45 µm filter and was loaded onto a His-Trap HP column. N- C- His-tagged protein was eluted by the mixture of buffer A (50 mM HEPES, pH 8.0; 500 mM KCl; 10% glycerol) and buffer B (50 mM HEPES, pH 8.0; 500 mM KCl; 10% glycerol; 500 mM imidazole) with a linear gradient of 10–100% (v/v).

TEV protease and 1 mM DTT were added in the collected fractions to cleave N-terminal His-tag. The amount of TEV protease used was 1.64 µg per 1 mg sample protein. The collected fractions were dialyzed out using N-terminal His-tag against buffer C (50 mM HEPES, pH, 8.0; 500 mM KCl; 10% glycerol; 1 mM DTT) overnight at 293 K. Subsequently, the dialyzed sample was loaded onto a His-Trap HP column again and C-His-tagged proteins was eluted by the mixture of buffer A and buffer B with a linear gradient of 4–40% (v/v).

Fractions containing target protein were further purified by gel filtration on a Hi-load 16/60 Superdex 200 column (GE Healthcare) with buffer D (50 mM HEPES, pH 8.0; 150 mM KCl; 10% glycerol) for BcsC-TPR domain and BcsC-TPR(N6), and with buffer E (50 mM HEPES, pH 8.0; 100 mM KCl) for BcsC-TPR (containing Asp24–Leu664 residues). Selenomethionine-substituted BcsC-TPR(N6) was purified using the same protocol as that of native BcsC-TPR(N6).

### Limited proteolysis analysis

Since purified C-His-tagged TPR domain (Asp24–Arg784) was sequentially degraded during purification and crystallization, we tried to find the stable parts of BcsC-TPR domain using the limited proteolysis analysis. The purified protein was mixed with trypsin on ice at a molar ratio of 100–100,000 to 1. After 0, 30, or 120 min, the mixed samples were checked using SDS-PAGE and analyzed using CBB staining (Fig. S3). The two strongest bands that appeared, were analyzed by N-terminal amino acid sequencing (at the global facility center at Hokkaido University) and matrix-assisted laser desorption/ionization-time of flight-mass spectrometry (MALDI-TOF-MS). Prior to MALDI-TOF-MS, the purified protein was diluted with acetonitrile. The final concentrations of protein and acetonitrile were 25 pmol mL^−1^ and 50%, respectively. After dilution, 0.65 µL protein sample and 1.3 µL matrix buffer (0.1% triflouroacetic acid and 10 mg mL^−1^ sinapinic acid in 50% acetonitrile) were loaded on the gold plate. Molecular weight was measured by Ultraflex-S (Bruker Daltonics).

### Crystallization and data collection

Purified BcsC-TPR(N6) was concentrated to 10 mg mL^−1^ by Vivaspin MWCO: 10,000 (GE Healthcare) and dialyzed against buffer F (50 mM Tris, pH 8.0; 150 mM NaCl) for crystallization. BcsC-TPR(N6) was crystallized using the sitting vapor diffusion method at 293 K using 96-well plates. A solution of BcsC-TPR(N6) was mixed with the reservoir solution and the ratio of protein to reservoir solution was 0.5 µL to 0.5 µL. Crystallization screening was carried out using crystallization kits (JCSG Core Suite I, II, III, IV, JCSG+ Suite, PACT Suite, PEGs Suite, PEGs II Suite, pHClear Suite, and pHClear II Suite produced by Qiagen). The initial crystals were obtained in the same condition containing 0.1 M MES, pH 6.0, and 4 M NaCl. The condition was optimized by changing the protein concentration, the ratio of protein solution to reservoir, the pH of MES, and the concentration of NaCl. Finally, the best crystals were grown under conditions of 60 mg mL^−1^ protein concentration, and a 0.5–1.0 µL ratio of protein solution to reservoir containing 0.1 M MES, pH 6.2, and 3.5 M NaCl (Table S2, Figs S6 and S7). Se-BcsC-TPR(N6) was crystallized using the sitting vapor diffusion method at 293 K. The initial crystals were obtained under the same conditions as native BcsC-TPR(N6). Optimized Se-BcsC-TPR(N6) crystals were obtained with 30 mg mL^−1^ protein concentration under conditions containing 0.1 M MES (pH 5.8) and 3.5 M NaCl.

The diffraction datasets of BcsC-TPR(N6) and Se-BcsC-TPR(N6) crystals were collected at beam line BL5A of Photon Factory (Tsukuba, Japan) and BL44XL of SPring8 (Harima, Japan), respectively (proposal no. 2014A1264 and 2015B1124). A wavelength of 0.979 Å was selected based on the fluorescence spectrum of Se absorption edge to measure the maximum anomalous scattering signal. Both crystals were soaked in the reservoir containing 20% glycerol, then flash-cooled to 93 K under a liquid nitrogen stream for data collection. All datasets were indexed, integrated, scaled, and merged with the XDS program^36^. Both crystals of native and selenomethionine-substituted BcsC-TPR(N6) belonged to space groups *P*2_1_ with cell parameters of *a* = 115.8 Å, *b* = 55.6 Å, *c* = 165.1 Å, and *β* = 95.6°, and *a* = 115.6 Å, *b* = 55.8 Å, *c* = 164.8 Å, and *β* = 95.6°, respectively. Details of the data collection and the process are shown in Table S3.

### Structure determination and refinement

No structural homologies were available for determining the structure of BcsC-TPR(N6) by molecular replacement, therefore, the phases were calculated using the single-wavelength anomalous diffraction method using Se atoms as the scatters at 3.4 Å resolution with Phenix.autosol^37^. Eighteen out of twenty selenium atoms were found in an asymmetric unit. The initial model structure (62% of total residues in five BcsC-TPR(N6) molecules) was built with phase improvement by Phenix.autobuil^38^. Subsequently, structural refinement was performed using native data at 3.27 Å resolution. Completion of the structure was straightforward using iterative cycles of manual model fitting and rebuilding based on 2*F*_o_ − *F*_c_ and *F*_o_ − *F*_c_ electron density maps with program COOT^39^ following computational refinement with Phenix.refine^40^. We first completed the building of a model corresponding to chain C in the asymmetric unit, then the N-terminal 81 residues (Thr27–Leu108) and C-terminal 168 residues (Ser109–Phe271) of this model were superposed on other initial molecules, respectively. The details of refinement are shown in Tables S3 and S4.

### Structural analysis in solution by size exclusion chromatography-small-angle X-ray scattering (SEC-SAXS)

SEC-SAXS was used to analyze the structure of BcsC-TPR (containing Asp24–Leu664 residues) in solution. SEC-SAXS data collection was carried out under room temperature at the beamline BL-10C, Photon Factory (Tsukuba, Japan) with Alliance HPLC system (Waters). Sample was loaded onto a Superdex 200 Increase 10/300 GL column (GE Healthcare) with buffer E and the collected fractions were directly exposed to X-rays. A stainless steel cell with a light pass length of 1 mM and a 0.02 mM-thick quartz glass window was used as a sample cell. The fractional positions and molecular weight of BcsC-TPR were estimated by SEC with multi-angle static light scattering (MALS, DAWN HELEOS II (Wyatt)) using the same column before SEC-SAXS measurement. During data collection, the flow rate was set at 0.05 mL min^−1^ and a total of 120 X-ray scattering images were collected over 40 min. The scattering images were recorded on a PILATUS3 2M detector (Dectris) and each two-dimensional scattering image was circularly averaged to convert to the one-dimensional scattering intensity data by using SAngler software^41^. The scattering intensities were converted to an absolute scale using water as a standard^42^. The 15 images measured prior to the experiment were averaged to use as background data. Ultraviolet–visible absorption spectra were simultaneously measured with the SAXS cell using a fiber spectrophotometer QE65pro (Ocean Optics) to estimate sample concentrations of SAXS data in real time.

Every five SAXS profiles were averaged to improve the signal-to-noise ratio of the data, and the extrapolated scattering profile at a concentration of zero was calculated with the data in the descent side of the peak (frames 91–118) by ALMERGE^43^. The radius of gyration (R_g_) and the scattering intensity at zero angle (I(0)) were obtained from Guinier analysis under the condition of Guinier approximation, Q × R_g_ < 1.3 by AUTORG of ATSAS^44^. The pair distribution function, P(r), was calculated using GNOM^45^, and the maximum distance in the molecule (D_max_) was estimated based on P(r).

Twenty SAXS envelopes of BcsC-TPR were calculated from random dummy models using DAMMIF^46^. Continuously, an averaged envelope of 20 SAXS envelopes was generated by DAMAVER^47^ and then used as an initial model for final calculation of DAMMIN^48^. An atomic poly-Ala model of the BcsC-TPR super-helices with a crystal structure of BcsC-TPR(N6) was manually fitted to the SAXS envelope using PyMOL. The theoretical scattering profile of this model was calculated by CRYSOL^49^ in order to validate the modeling. Details of the result of SAXS experiment and analysis are summarized in Table S5.

## Electronic supplementary material

Supporting information
