# Supplementary material for: Crystal structure of the flexible tandem repeat domain of bacterial cellulose synthesis subunit C
Source: Sci Rep. 2017 Oct 12;7:13018. doi: 10.1038/s41598-017-12530-0 (PMC5638805; doi:10.1038/s41598-017-12530-0)
Supplement: Supplementary file 1 — Supporting information [file 41598_2017_12530_MOESM1_ESM.pdf]

Supporting information for

Crystal structure of the flexible tandem repeat domain of bacterial cellulose synthesis subunit C

Shingo Nojima,[1], Ayumi Fujishima,[1], Koji Kato,[1,2], Kayoko Ohuchi,[1], Nobutaka Shimizu,[3], Kento Yonezawa,[3], Kenji Tajima[4] , and Min Yao[1,2,*]

[1]Laboratory of X-ray structural biology, Graduate school of Life Science, Hokkaido University, Sapporo, Hokkaido, 060-0810, Japan

[2]Faculty of Advanced Life Science, Hokkaido University, Sapporo, Hokkaido, 060-0810, Japan

[3]Photon Factory, Institute of Materials Structure Science, High Energy Accelerator Research Organization (KEK), Tsukuba, Ibaraki, 305-0801, Japan

[4]Laboratory of Polymer Chemistry, Hokkaido University, Sapporo, Hokkaido, 060-8628, Japan

*Correspondence should to be addressed to M. Yao, email: yao@castor.sci.hokudai.ac.jp

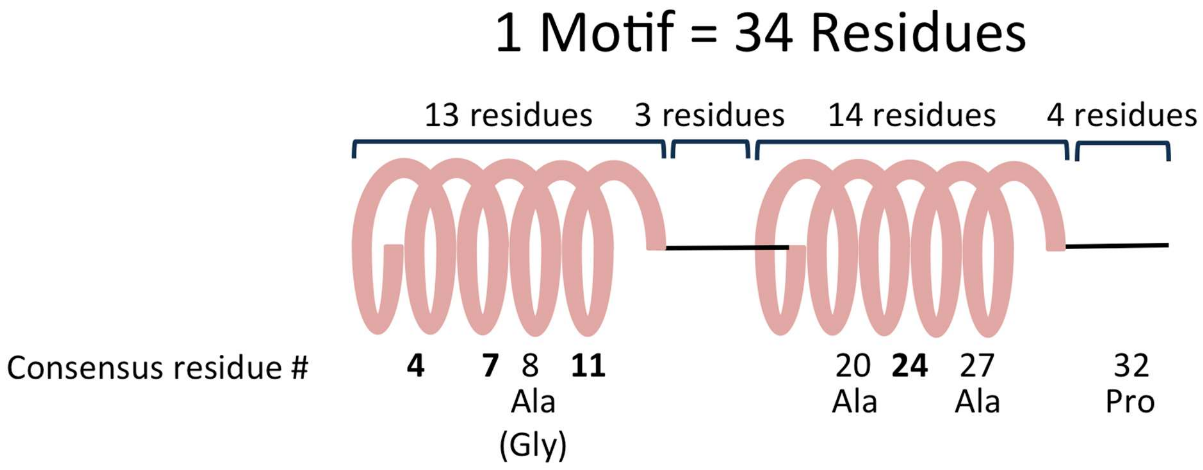

Figure S1 Secondary structure and consensus residues of the TPR motif. The TPR motif consists of two α-helices (13 and 14 residues) and two turns (3 and 4 residues). Conserved large hydrophobic amino acids were positioned at residue numbers shown in bold.

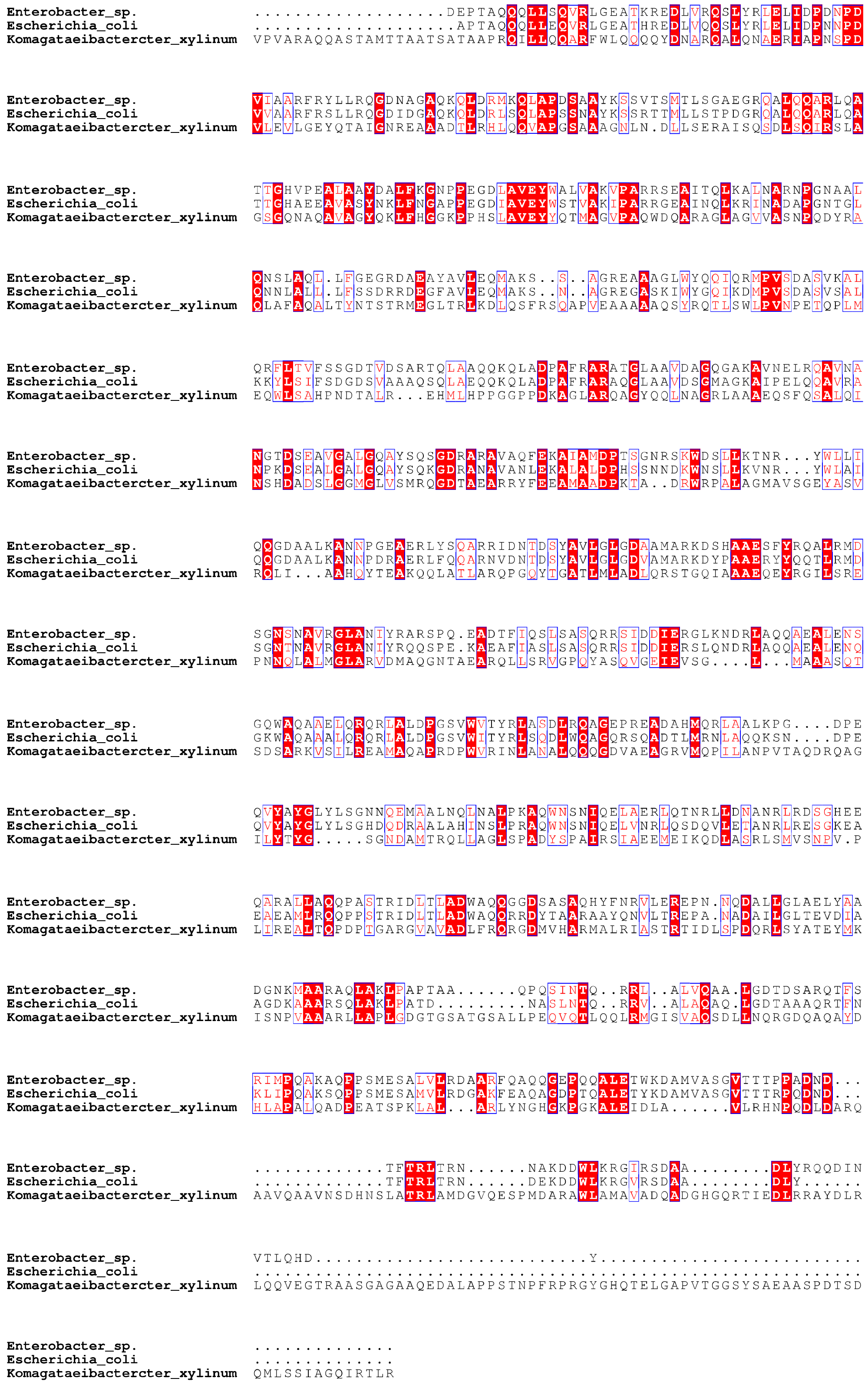

Figure S2 Conservation of BcsC-TPR domain. BcsC-TPR domains of *Enterobacter* CJF-002 (Enterobacter_sp.), *Escherichia coli* (Escherichia_coli), and *Komagataeibacter xylinum* (Komagataeibacter_xylinum) were aligned. Red boxes indicate strictly conserved region and red characters indicate similar regions.

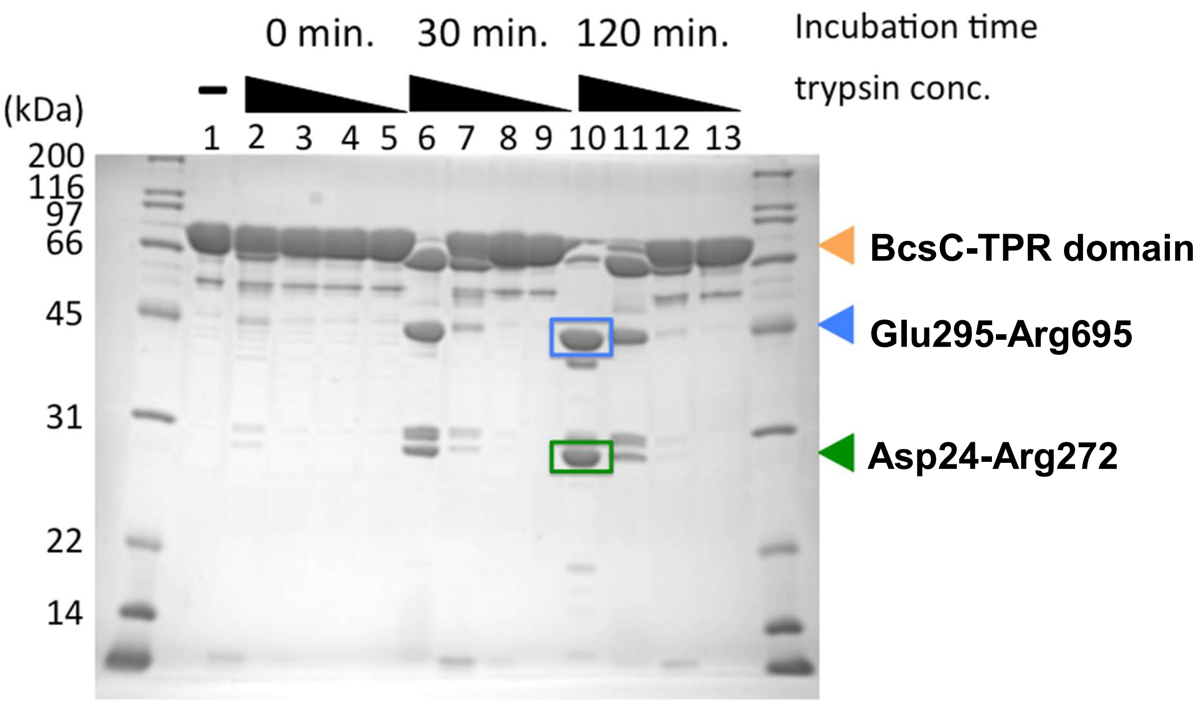

Figure S3 Limited trypsinolysis of BcsC-TPR domain (Asp24-Arg784). This figure shows 12.5 % SDS-PAGE gel stained by CBB. 100 μg BcsC-TPR domain was incubated with no trypsin (Lane 1), with 2 μg, 0.2 μg, 0.02 μg or 0.002 μg trypsin for 0 minute (Lane 2-5), with 2 μg, 0.2 μg, 0.02 μg or 0.002 μg trypsin for 30 minutes (Lane 6-9), with 2 μg, 0.2 μg, 0.02 μg or 0.002 μg trypsin for 120 minutes (Lane 10-13).

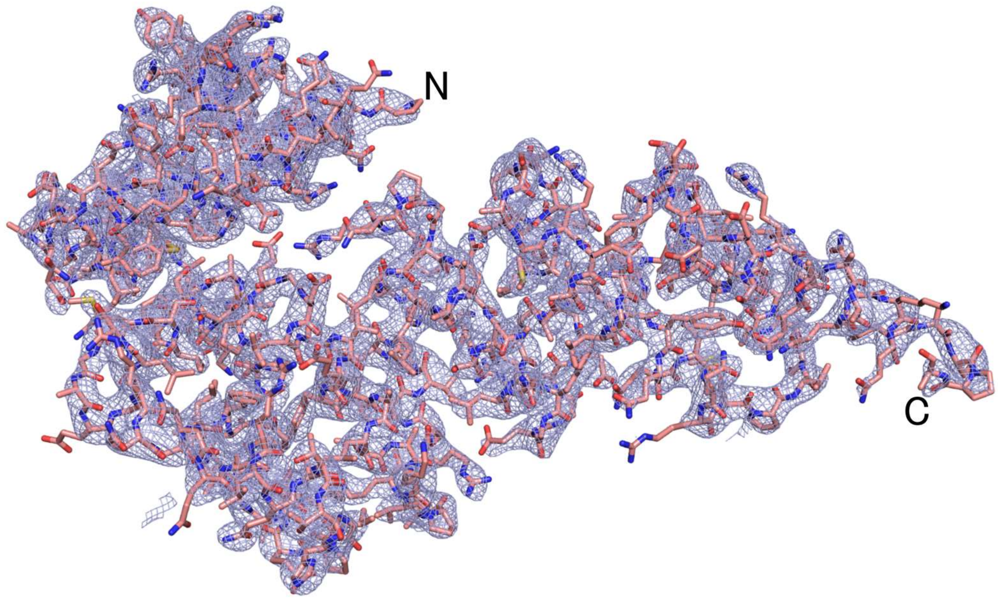

Figure S4 Electron density map of monomeric BcsC-TPR(N6). Monomeric BcsC-TPR(N6) are shown as wheat sticks. Electron density map contoured at 1.5 σ level are shown as blue mesh.

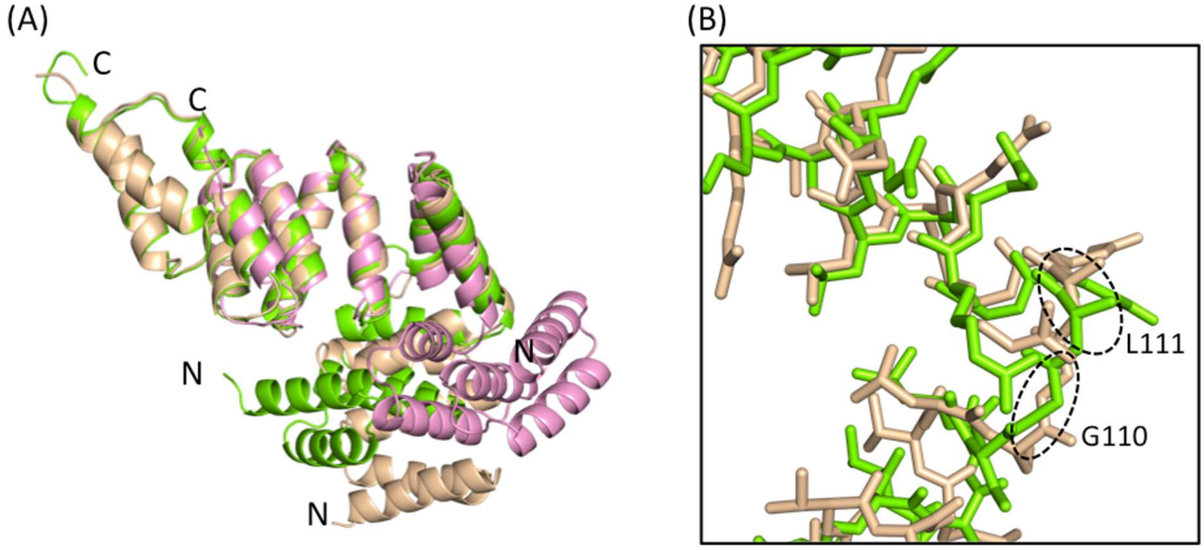

Figure S5 Structural comparison of BcsC-TPR(N6)s. (A) Superposition of three type chains on α6–α12. (B) Zoomed in and shown in stick format around the turn of inserted region.

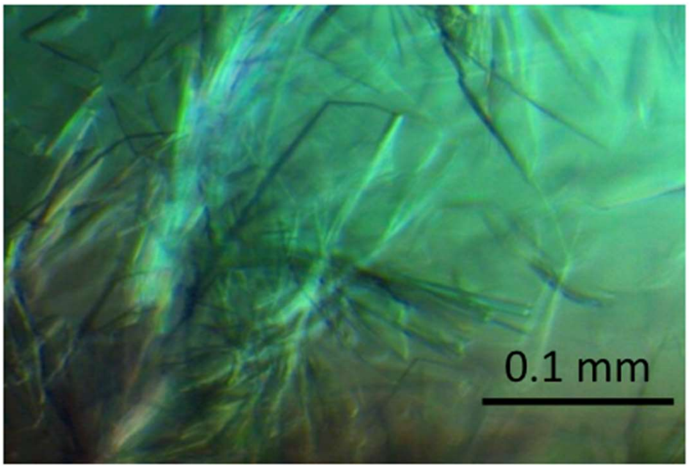

Figure S6 The crystal photo of BcsC-TPR(N6)

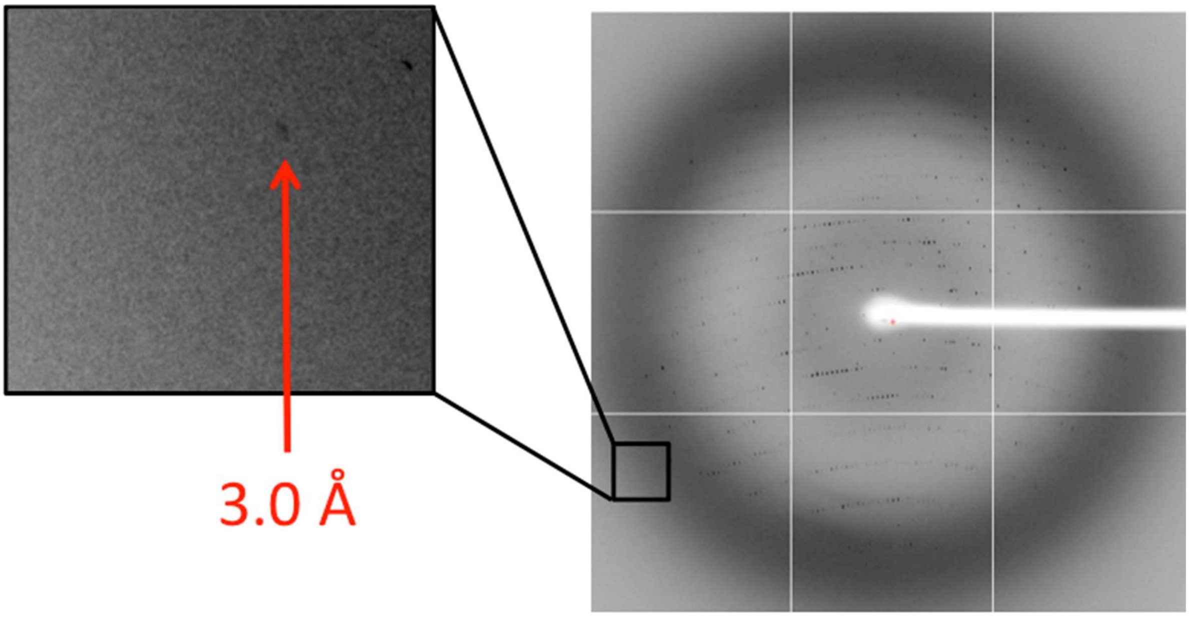

Figure S7 X-ray diffraction image of a crystal of native BcsC-TPR(N6). Right, X-ray diffraction image of a crystal of BcsC-TPR(N6); left; zoomed-in X-ray diffraction image. The red arrow indicates the observed diffraction spot at maximum resolution (3.0 Å).

Table S1 Macromolecule production information

	BcsC-TPR domain (Asp24-Arg784)	BcsC-TPR (Asp24–Leu664)	BcsC-TPR(N6) (Asp24–Arg272)
Source organism	*Enterobacter* CJF-002	*Enterobacter* CJF-002	*Enterobacter* CJF-002
DNA source	UniProt K0IS18	UniProt K0IS18	UniProt K0IS18
Forward primer	ACGGATCCGCATGCG (NdeI)	ACGGATCCGCATGCG (NdeI)	ACGGATCCGCATGCG (NdeI)
Reverse primer	CGATCCTCTCATAGTTA ATTTC (XhoI)	CCGCTCGAGTTATGCT TTCGCCTGCGG (XhoI)	CCGCTCGAGTTAGCGA AACGCCGGATCG (XhoI)
Cloning vector	Modified pET28a	Modified pET28a	Modified pET28a
Expression vector	Modified pET28a	Modified pET28a	Modified pET28a
Expression host	BL21 (DE3)	BL21 (DE3)	BL21 (DE3)

Table S2 Crystallization

Method	Sitting vapor-diffusion
Plate type	NeXtal Evolution µplate (QIAGEN)
Temperature (K)	293
Protein concentration	60 mg / mL
Buffer composition of protein solution	50 mM HEPES (pH 8.0), 150 mM KCl, 10 % glycerol
Composition of reservoir solution	100 mM MES (pH 6.2), 3.5 M Sodium chloride
Volume and ratio of drop	0.5 µL + 0.5 µL
Volume of reservoir	50 µL

Table S3 Data collection and processing statistics

	Native BcsC-TPR(N6)	SeMet BcsC-TPR(N6)
Diffraction source	BL5A, Photon Factory	BL44XU, Spring8
Wavelength (Å)	1.0	0.979
Temperature (K)	100	100
Detector	ADSC Quantum 315r (CCD)	RAYONIX MX300HE (CCD)
Crystal-detector distance (mm)	419	350
Rotation range per image (°)	0.5	1
Total rotation range (°)	180	360
Exposure time per image (s)	2	0.3
Space group	$P2_1$	$P2_1$
a, b, c (Å)	115.8, 55.6, 165.1	115.6, 55.8, 164.8
α, β, γ (°)	90, 95.6, 90	90, 95.6, 90
Resolution range (Å)	50 – 3.27 (3.47–3.27)	50 – 3.4 (3.6-3.4)
Total No. of reflections	121528	216119
No. of unique reflections	32542	56065
Completeness (%)	98.6 (98.2)	99.4 (96.8)
Redundancy	3.7 (3.8)	3.85 (3.82)
$\langle I/\sigma(I)\rangle$	10.7 (2.0)	8.40 (2.25)
Rmeas † (%)	13.4 (80.3)	24.3 (113.1)
Rmerge ‡ (%)	11.5 (69.0)	20.9 (97.0)
Overall B factor from Wilson plot (Å^2)	62.5	60.6

†$R_{\text{meas}} = \Sigma_{hkl}\{N(hkl)/[N(hkl)-1]\}^{1/2}\Sigma_i|I_i(hkl) - \langle I_{hkl}\rangle|/\Sigma_{hkl}\Sigma_i I_i(hkl)$, where $I_i(hkl)$ and $N(hkl)$ are the mean intensity of a set of equivalent reflections and the multiplicity, respectively.

‡$R_{\text{merge}} = \Sigma_{hkl}\Sigma_i|I_i(hkl) - \langle I(hkl)\rangle|/\Sigma_{hkl}\Sigma_i I_i(hkl)$, where $I_i(hkl)$ is the *i*th observation of reflection *hkl* and $\langle I(hkl)\rangle$ is the average intensity for all *i* observations of reflection *hkl*.

Table S4 Structure solution and refinement statistics

Resolution range (Å)	47.7 – 3.3
Completeness (%)	98.7
No. of reflections, working set	30914
No. of reflections, test set	1627
Final R_{cryst}	0.21
Final R_{free}	0.26
No. of non-H atoms	
Protein	8741
R.m.s. deviations	
Bonds (Å)	0.01
Angles (°)	1.40
Average B factors (Å^2)	
Protein	82.7
Ramachandran plot	
Most favoured (%)	97
Allowed (%)	2.7

Table S5 Details of the result of SAXS experiment and analysis.

Data-collection parameters	
Beamline	PF BL-10C
Beam geometry Beam size	Bent cylindrical mirror + Two slits V0.35×H0.55mm
Wavelength (Å)	1.000
Camera distance (mm)	2011
q range ($Å^{-1}$)	0.008 – 0.420
Exposure time (sec)	20
Temperature (K)	293
Structural parameters	
I(0) (cm^{-1}) [from P(r)]	0.104 ± 0.0003
R_g (Å) [from P(r)]	51.3 ± 0.2
I(0) (cm^{-1}) [from Guinier]	0.105 ± 0.0004
R_g (Å) [from Guinier]	51.2 ± 0.8
D_{max} (Å)	185.3
Porod volume estimate ($Å^3$)	115373
Molecular-mass determination	
Estimated MW [from Porod vol.] (kDa)	72.1
Calculated MW from sequence (kDa)	71.3
Estimated MW [from SEC-MALS] (kDa)	71.4
